# Endocrinology of Underweight and Anorexia Nervosa

**DOI:** 10.3390/nu15163509

**Published:** 2023-08-09

**Authors:** Christian Fricke, Ulrich Voderholzer

**Affiliations:** 1 Schoen Klinik Roseneck, 83209 Prien am Chiemsee, Germany; uvoderholzer@schoen-klinik.de; 2 Klinik für Psychiatrie und Psychotherapie, Ludwig Maximilians Universität, 80336 Munich, Germany

**Keywords:** anorexia nervosa, endocrinology, constitutional thinness, hormones, hormonal alterations

## Abstract

More than any other mental illness, the course, prognosis, and therapy of anorexia nervosa are shaped by the physical changes associated with being underweight. This article provides an overview of the endocrine changes associated with malnutrition and underweight. This overview serves as a basis for understanding the other articles in this special issue, which deal with the health risks associated with being underweight. In this context, the differences between underweight in anorexia nervosa and in constitutional thinness are of particular importance in assessing the impact of intentional weight loss. In this context, the regulation of hunger and satiety deserves special interest, as this is the area in which the intentional influence on body weight comes into play. Clinical consequences on, for example, fertility, bone metabolism, the homeostasis of, for example, serum glucose levels, or body temperature have been observed for a long time; nonetheless, the medical responses, apart from vitamin supplementations and advice to gain weight, are still limited. Therefore, emphasis was placed on the potential improvement of outcomes through the administration of central or peripheral hormones. Studies were identified on PubMed via a selection of relevant keywords; original texts that were cited in reviews were studied where it was advantageous. This review found some promising data on bone health and the administration of transdermal oestrogen, which is not yet widely used, as well as distinct hormonal markers to differentiate between CT and AN. We concluded that the continuous efforts to investigate the role of endocrinology in underweight and/or anorexia nervosa lead to outcome benefits and that more and higher-powered studies are needed.

## 1. Introduction

Eating disorders are mental illnesses that, much more clearly than other mental illnesses, are characterised in their course, treatment, and prognosis by those changes that are marked by the physical consequences of the eating disorder. In this context, the more significant part of the ultimate consequences is due to the food restriction, the lack of energy and, ultimately, the excessively low body weight. In this respect, the findings are particularly valid for anorexia nervosa and somewhat less so for other eating disorders, such as bulimia nervosa or binge eating disorder.

The endocrine consequences that will be reviewed in this review affect the pituitary gland, hypothalamus, adrenal glands, gonads, and brain–gut axis. By looking at the difference between constitutional thinness and anorexia nervosa, it becomes clear which of the endocrine changes are due to the body’s face itself and which are due to food restriction and mental disturbance.

## 2. Methods

This review article identified studies via PubMed using keywords and the criterion of associations between anorexia nervosa or underweight and endocrine alterations. In some cases, meta-analytic papers were cited instead of the original texts if they were either from the 20th century or their results had been already widely discussed.

The following hormones were reviewed: pituitary hormones and the consequentially released peripheral hormones, as well as hormones regulating appetite.

In order to distinguish between symptoms and alterations following restricted food intake as opposed to underweight due to other reasons, a second section concerning studies on constitutional thinness, anorexia nervosa, and their respective endocrine alterations, all found on PubMed, was reviewed.

The field of research is too large for a systematic review; therefore, for this editorial, between early 2022 and mid-2023, we selected sources using the following mesh terms:

(“Feeding and Eating Disorders” [MeSH Terms] OR “Binge-Eating Disorder” [MeSH Terms] OR “Feeding and Eating Disorders of Childhood” [MeSH Terms] OR “Anorexia Nervosa” [MeSH Terms] OR “Bulimia Nervosa” [MeSH Terms]) AND (((“Ghrelin” [MeSH Terms] OR “Leptin” [MeSH Terms]) AND “Peptide YY” [MeSH Terms]) OR (“Glucagon” [MeSH Terms] OR “Glucagon-Like Peptide 2” [MeSH Terms] OR “Glucagon-Like Peptide 1” [MeSH Terms]) OR (“Diabetes Mellitus” [MeSH Terms] OR (“Glucose Metabolism Disorders” [MeSH Terms] OR “Glucose Intolerance” [MeSH Terms]) OR (“Thyroid Gland” [MeSH Terms] OR “Thyroid Hormones” [MeSH Terms] OR “Thyroid Function Tests” [MeSH Terms] OR “Thyroid Diseases” [MeSH Terms]) OR (“Adrenal Glands” [MeSH Terms] OR “Epinephrine” [MeSH Terms] OR “Pituitary–Adrenal System” [MeSH Terms] OR “Pituitary–Adrenal Function Tests” [MeSH Terms] OR “Adrenal Medulla” [MeSH Terms] OR “Adrenal Cortex Hormones” [MeSH Terms]) OR “Norepinephrine” [MeSH Terms] OR “Growth Hormone” [MeSH Terms] OR “Hormones” [MeSH Terms] OR (“Growth Hormone-Releasing Hormone” [MeSH Terms] OR “Pituitary Hormone-Releasing Hormones” [MeSH Terms] OR “Gonadal Hormones” [MeSH Terms] OR “Pituitary Hormones” [MeSH Terms] OR “Hypothalamic Hormones” [MeSH Terms] OR “Gonadal Steroid Hormones” [MeSH Terms]) OR (“Insulin-Like Growth Factor I” [MeSH Terms] OR “Insulin-Like Growth Factor II” [MeSH Terms]))).

Articles were included if they appeared in the results list and were relevant to either the diagnostics, outcome, or therapy of either anorexia nervosa or constitutional thinness. There were no formal exclusion criteria.

## 3. Results

The Diagnostic and Statistical Manual of Mental Disorders, fifth edition (DSM-5), criteria for AN define “low body weight” and “restriction of energy intake relative to requirements” as two of its main characteristics. This state of semi-starvation results in several hormonal adaptations aiming at survival, but partly leading to collateral pathologies that require medical attention, such as bone metabolism and fertility. After describing the observed alterations in the hypothalamic–pituitary axis and satiety regulation, followed by a comparison with endocrine changes in CT, this chapter will review the role of hormone supplementation according to exemplary studies selected according to their perceived clinical relevance (Table 1).

### 3.1. Hypothalamic-Pituitary Dysfunction in AN

The pituitary gland or hypophysis is a pea-sized endocrine gland protruding caudally of the hypothalamus and sitting in the sella turcica. It can be functionally divided into an anterior and an intermediate part, whose endocrine cells are regulated by hypothalamic-releasing hormones, and a posterior part that stores and secretes hormones produced in the hypothalamus.

Some of the body processes regulated by the pituitary gland are closely linked to the starving state in AN, as they concern the metabolic conversion of food into energy, water and osmolarity regulation, temperature regulation, growth, and reproductive health. Therefore, each hormone axis, its physiological effect, and adaptive alteration will be regarded separately after the following overview of hormones concerned.

The hormones produced and/or secreted in the respective lobes are:

Anterior:
  - Thyroid-stimulating hormone (TSH);
  - Adrenocorticotropic hormone (ACTH);
  - Follicle-stimulating hormone (FSH) and luteinizing hormone (LH);
  - Growth hormone (GH);
  - Prolactin (PRL).
Intermediate:
  - Melanocyte-stimulating hormone (MSH).
Posterior:
  - Antidiuretic hormone (ADH);
  - Oxytocin (OXT).

#### 3.1.1. Hypothalamic-Pituitary-Thyroid Axis

The effects on the body of the thyroid hormones triiodothyronine (T3) and thyroxine (T4) include, among many others, cardiovascular and metabolic effects that increase resting energy expenditure. In starvation, the concentrations of primarily T3 and sometimes also T4 are, therefore, regulated down in order to preserve energy [1,2,3,4,5], whereas TSH is usually normal, with some variations. During recovery, i.e., weight gain, these alterations are reversible.

Clinically, these hypothyroid alterations result in, or co-factor, typical symptoms of AN, such as bradycardia, decreased appetite, constipation, cold intolerance, and hair loss. Another typical symptom of hypothyroidism, weight gain, is outplayed by restrictive food intake and sometimes exercise.

While these symptoms in a hypothyroid patient would prompt doctors to substitute thyroid hormones in order to avoid these disturbances, in patients with AN, it is important to regard the sequence of cause and effect of low-T3 Syndrome. As, in anorexia nervosa, the hypothyroid metabolic state is the response to a lack of energy, the supplementation of thyroxine would result in yet aggravated energy deficiency and, therefore, be potentially fatal. Furthermore, the risk of abuse of thyroxine is substantial, as increasing energy consumption is one of the maladaptive behaviours secretly adopted, especially during therapy where food intake and exercise are monitored [6].

Nonetheless, checking for underlying thyroid abnormalities has its role in the diagnostic process of AN, as hyperthyroidism can be a cause of weight loss and hypothyroidism could result in anorectic behaviour as a consequence of the endocrine weight gain.

#### 3.1.2. Hypothalamic-Pituitary-Adrenal (HPA) Axis

ACTH is released alongside beta-endorphin and alpha-MSH, which are also cleaved from the precursor Pro-opiomelanocortin (POMC) under the influence of corticotropin-releasing hormone (CRH), which is secreted in the hypothalamus. ACTH stimulates adrenal cortex cells to secrete glucocorticoid steroid hormones, which are simplistically regarded as “stress hormones” and whose effects of concern in AN are mainly immunological and metabolic. While down-regulating inflammatory processes, endogenic glucocorticoids also serve to maintain blood glucose levels via various metabolic effects, including gluconeogenesis, inhibition of glucose uptake, and providing a substrate for gluconeogenesis from amino acids and fatty acids.

In AN, high levels of ACTH and cortisol can be observed, indicating a chronically stimulated state of the HPA axis [7,8]. Also, the cortisol awakening response (CAR) is elevated. In AN, the inverse correlation of hypercortisolaemia and body mass index (BMI) indicates a causal relationship, the hypothesis for which is the increased need for maintaining euglycemia in a state of starvation [9].

The equivalent clinical syndrome of hypercortisolaemia in non-anorexic patients would be Cushing syndrome; albeit being clearly distinct in appearance, it may still be of interest to compare their clinical parallels. In both diseases, the elevated cortisol levels have a disadvantageous effect on bone mineral density (BMD), immunological function, insulin resistance, and trunk fat accumulation, which, in AN, can be observed during weight recovery when the baseline cortisol levels are high [10].

Due to these adverse effects of hypercortisolaemia, pharmacological interventions could be considered at first sight, but are not recommended due to, again, the adaptive nature of this endocrine response in order to preserve euglycaemia and normotension [11].

#### 3.1.3. Hypothalamic-Pituitary-Gonadal (HPG) Axis

The pituitary hormones LH and FSH are stimulated by the hypothalamic gonadotropin-releasing hormone (GnRH), which is regulated down in the state of chronic starvation. Subsequently, lower LH pulsatility, as well as reduced levels of oestradiol and testosterone, can be observed [12] in females. Clinically, this can result in secondary amenorrhea, originally a diagnostic criterion for AN, but was dropped in the DSM-5 with regard to the variability in endocrine dysfunction in AN (and the inclusion of male patients): some women meet the criteria of restrictive energy intake and significantly low weight, intense fear of gaining weight or persistent behaviour that interferes with weight gain, and disturbance in the perception of one’s body weight or shape without losing neuroendocrine control of reproductive function [13]. On the other hand, various studies have shown amenorrhea to be persistent after weight recovery in significant numbers of women formerly meeting all the criteria of AN, resulting in the psychologically stressful possible long-term condition of infertility [14,15].

Testosterone and oestrogen are also important for skeletal integrity, which will be reviewed later on in this chapter, muscle maintenance, as well as in anxiety and depression. While it may seem sensible from an evolutionary point of view to pause the reproductive system during chronic starvation, the negative effects on BMD and reproductive functions have instigated numerous studies on, mostly, oral hormone replacement. For many years, these produced insufficient data to support supplementation, the hypothesis being that oral oestradiol suppresses the hepatic synthesis of insulin-like growth factor 1 (IGF-1), which would offset the desired effect. Some studies using transdermal oestrogen support the idea of avoiding this first-pass effect and showed increased bone accrual rates in adolescents with AN, and should be recommended alongside oral gestagen supplementation [16].

Sex hormone deficiency can result in neuropsychiatric disturbances such as anxiety and depression, and could therefore arguably aggravate these conditions in the presence of AN. While physiological transdermal oestrogen replacement did have an easing effect on anxiety symptoms in adolescent girls, it did, however, not change the core AN features of restrictive eating, nor perception of one’s body weight or shape [17].

#### 3.1.4. Growth Hormone—IGF-1 Axis

The endocrine adaptation to chronic fasting or starving also includes a state of GH resistance, in which elevated levels of GH due to elevated stimulation by Ghrelin can be observed alongside reduced levels of IGF-1. Again, this resistance takes place at the level of the liver and might be an adaptation to the energy deficiency in order to decrease energy consumption by growth [18,19]. As GH also has a role in gluconeogenesis, partly through the mobilization of fatty and amino acids and, thus, is similar to the effect of glucocorticoids, the hypothesis is that the primary adaptive process is the up-regulation of GH in order to preserve euglycaemia. Secondary to this, the liver regulates GH effects down in order to save energy, which would otherwise outdo this adaptation by using the provided energy for growth. Attempts to explain this in endocrine terms have included the regulatory effect of fibroblast growth factor 21 (FGF21) and insulin, whilst low IGF-1 levels and elevated ghrelin levels stimulate GH secretion. During recovery, the IGF-1 levels increase with refeeding, while the GH levels normalize with weight gain [8,20,21].

The effects of GH resistance include reduced height, as late puberty reductions of GH correlate with the closure of epiphyseal plates, but there was insufficient proof of girls benefitting from GH administration in terms of longitudinal bone growth. Attempts to substitute recombinant GH did not increase IGF-1 levels, and the lipolytic effects of GH in underweight patients are considered adverse [22].

#### 3.1.5. Prolactin

There is no evidence that prolactin levels are significantly altered in patients with AN, unless treated with psychotropic medication affecting dopamine receptors; hence, there is no clinical importance of prolactin, nor its pharmacological administration in this context.

#### 3.1.6. Anti-Diuretic Hormone (ADH)

ADH is secreted by the posterior pituitary in response to hypertonicity and leads to the reabsorption of water in the distal and cortical collecting tubules and the concentration of urine. The syndrome of inappropriate ADH secretion (SIADH) results in hyponatraemia, which, itself, is common in AN and can lead to serious adverse symptoms, such as vomiting, confusion, or seizures. However, there is more to be said about hyponatraemia for different reasons to the effect of ADH, as excess drinking of water to manipulate weight is a major risk [23,24]. Therefore, close care, both medical and therapeutic, needs to be taken in the case of low sodium levels, and the administration of SSRIs in AN should be considered carefully as SIADH is one of their frequent adverse effects, and evidence for its use in severely underweight patients is scarce [25].

#### 3.1.7. Oxytocin

Oxytocin (OXT) is a hormone associated with social bonding and reproduction, and plays a role during and after childbirth, as well as in anxiety and depression. OXT is altered in patients with AN, as its peripheral levels during the night are lower in patients with AN than those in the normal population, but higher postprandially or in weight-recovered patients with AN [26,27,28,29]. The reason for this can possibly be found in the inverse correlation of the peripheral and central OXT levels.

There is no evidence for a therapeutic effect of oxytocin administration in patients with AN.

### 3.2. Bone Metabolism

Reduced skeletal integrity is an important physical consequence of AN and has been the subject of numerous studies. The accordingly elevated fracture rate can be seen, even when BMD is normal or close to normal, which suggests that the microarchitecture of the bone is an independent factor on top of bone mass density, as measured radiologically [30,31,32]. Reduced BMD is not a long-term effect of AN, but can be observed fairly early in the course of the disease.

The most important endocrine factors contributing to bone loss are IGF-1 deficiency, secondary to GH resistance, oestrogen and testosterone deficiency, and excess cortisol [33]. To a lesser extent of proof in humans, hyponatremia, reduced oxytocin and leptin, and elevated levels of peptide YY are associated with a reduction in BMD.

The main cure remains the restoration of weight and menstrual function, but there is a place for pharmacotherapy attempting to maintain BMD. Whilst oral oestrogen and bisphosphonates have not proven to be beneficial and vitamin D and calcium alone are insufficient for this goal, physiological transdermal oestrogen with cyclic progesterone prevented the decrease in BMD observed in the control group [34,35,36].

### 3.3. Regulation of Food Intake

At their core, eating disorders are disorders of the regulation of food intake, i.e., disorders of hunger, satiety, and fullness. A prerequisite for food intake that is appropriate to the body’s needs is communication between the organism and the centres in the hypothalamus that regulate food intake. In the last thirty years, we have learned many new things about the transmission of this kind of information from the periphery to the hypothalamus. Here, the adipocytes and the gastrointestinal tract play a central role.

Adipokines are a group of endocrine-active proteins from fat cells. Of the variety of these regulators, only adiponectin and especially leptin, which was the very first of these adipokines to be discovered in 1994, seem to be of importance for food intake.

#### 3.3.1. Adiponectin

Adiponectin is involved in the regulation of glucose and fatty acid oxidation. It is a protein secreted by adipose tissue and is inversely related to BMI. As in other forms of underweight and cachexia, adiponectin is elevated in anorexia [37,38,39,40,41,42] and has been reported to be lower, similar, or higher in other forms of eating disorders compared with controls [38,43] and decreases with refeeding, as expected [44]. The role of adiponectin in the regulation of appetite and food intake remains unclear [45].

#### 3.3.2. Leptin

Leptin was discovered when looking for the cause of obesity in genetically significantly overweight mice [46]. The reason is that these mice genetically lack the ability to release leptin, a peptide hormone released by fat cells that inhibits appetite and food intake once the fat cells have stored sufficient energy in the form of fat. In anorexia nervosa, extremely low leptin levels are found, corresponding to the almost completely depleted adipocytes [47,48,49]. Nevertheless, this does not lead to an increase in food intake in those affected. In contrast, the increased urge to exercise in many of those affected seems to be due to the almost complete absence of leptin in the bloodstream. In mice in a state of hunger, an increased urge to move can regularly be observed, which phylogenetically probably serves to search for food. This increase in physical activity can be antagonised by the exogenous supply of leptin, which is low in the starvation state [50,51].

In AN, excessive exercise is common [52,53,54,55,56] and might be partly due to this phenomenon. Leptin might also have a role in amenorrhea in AN, as it stimulates GnRH [57,58]. Recent data derived from observations with small numbers of patients suggest a positive correlation of leptin levels with outcome following treatment, as well as a promising potential of the administration of metreleptin in AN [59,60,61,62,63].

While it is evident that increased physical activity is an important issue in the treatment of many patients with anorexia nervosa [52,64,65], it remains unclear to date how much treatment outcomes are affected by physical activity and whether treatment programs that specifically address physical activity have an advantage over standard treatment approaches [56,66,67]. However, a controlled study demonstrated that a specific group intervention can reduce the urge to exercise [68].

#### 3.3.3. Cholecystokinin (CCK)

CCK is produced in the gastrointestinal tract, in the upper sections of the small intestine, and plays an important role in activating digestion after food intake. At the same time, it regulates food intake so that increasing satiety is induced by its release and the amount of food ingested is thus controlled. However, this limitation of meal size has no effect on weight control. Binge eating excessive amounts of food, as observed in the binge eating disorder, bulimia nervosa, and in binge–purging-type anorexia nervosa, are typically accompanied by decreased CCK release. As a result, those affected lack the feeling of satiety [69,70,71,72].

#### 3.3.4. Peptide YY (PYY)

PYY is released in the ileum and colon in response to feeding. PYY reduces appetite and is thus considered an anorexigenic, partly by reducing the effect of ghrelin. Therefore, reduced serum levels of PYY would be expected in patients with AN. However, in some studies, they have been reported to be significantly higher than those in the control group [73,74,75,76,77]. The levels of PYY may also remain elevated, even after weight restoration [78]. It is, therefore, believed that this is not an adaptive process, but a factor independent of eating behaviour contributing to restrictive eating in AN due to a loss of appetite.

#### 3.3.5. Ghrelin

Ghrelin, the only orexigenic circulating factor known until now and sometimes referred to as the “hunger hormone”, is elevated in AN and decreases with weight restoration [79,80,81,82]. Ghrelin is a stronger stimulus for GH than GHRH. In increasing appetite, there could be a role in the treatment of AN, but so far, studies have been of limited power [83,84].

### 3.4. Anorexia Nervosa vs. Constitutional Thinness (CT)

Constitutional thinness is a condition which is ill-defined, but most commonly referred to as a state of severely reduced weight in the absence of eating disorders, as well as associated pathologies. Hormonal alterations are not considered typical for CT, especially because, in contrast to the criteria of AN in DSM-IV, reproductive function is not impaired in CT. As amenorrhea is no longer a diagnostic criterion for AN in DSM-5, the distinction between the two has become somewhat more difficult to make, for the difference is now, besides behavioural observations where these are possible, partly a subjective psychological criterion subject to individuals’ adherence to telling their mind. It has been postulated that part of the statistical rise in AN is due to misdiagnoses of CT as AN since the implementation of DSM-5 criteria. Therefore, a subchapter on this subject is of interest as there are, in opposition to outdated comments on CT bearing no endocrine abnormalities, hormonal aspects that can help to distinguish the two conditions (Table 2). The following paragraphs will look at mostly, but not only, endocrine similarities, showing that CT should not be mistaken for physiological thinness, and differences, proving that there is indeed a clear distinction.

#### 3.4.1. Similarities of AN and CT

Individuals are considered constitutionally thin when their BMI is below 17.5 kg/m² but they are neither over-exercising, undernourished, suffering from eating disorders, nor from diseases causing cachexia. It, therefore, comes as no surprise that the eye-catching resemblance of the two conditions is in fact being underweight, although not as severely in CT when compared with AN. Beyond the obvious, AN and CT both exhibit similar values for fat-free mass (FFM) and, more importantly, for bone mass density (BMD) [85,86,87]. CT individuals, therefore, are underweight, but not underfat and may be at higher risk of bone fractures. It is, therefore, possible that being constitutionally thin has an effect on health. However, there is a lack of data on this.

#### 3.4.2. CT in between AN and Control

As FFM is similar in CT and AN, but BMI is higher in CT than in AN, fat mass (FM) must also be higher in CT. Although both the differences in FM in AN vs. CT and CT versus the control group were significant, %FM in CT was within the normal range of body fat [85]. Leptin was also observed to be blunted in CT, but to a lesser extent than in AN, while the results for ghrelin were meta-analytically difficult to assess due to the different methods used when measuring ghrelin. The resting metabolic rate, as an indicator of energy expenditure, has been subject to a large number of studies with equivocal results, but the meta-analysis showed the same significant differences as those in BMI and FM [88]. When looking at the ratio of RMR/FFM, the difference becomes even more pronounced, suggesting a metabolically highly active fat-free mass in CT, and hypothesizing this to be a factor in weight gain resistance [85,89].

#### 3.4.3. AN Differing from CT and Control

Energy intake can be measured, and is, by definition, lower in AN than in CT and control groups, where no food restriction is observed [90]. This constitutes the most important non-subjective difference between the two conditions. There are, however, other differences in line with the original distinction of CT not having any effect on reproductive health. Estradiol, FSH, and LH are found to be normal in CT, whilst being significantly blunted in AN [89,91]. As the same result has been found for IGF-1 and GH [89], there remains a puzzle as to which factor the aforementioned reduced BMD in CT should be attributed.

#### 3.4.4. AN and CT Diametrically Opposed

When looking for endocrine distinctions to separate CT from AN, the first factor to be remembered is fT3, which is significantly elevated in CT vs. the control, although in clinical terms, this difference is so slim that it could only be observed in a meta-analysis. However, as fT3 in AN is severely blunted, this remains a marked distinction, whilst giving a possible explanation for the conspicuous RMR/FFM ratio in CT [85,89].

Furthermore, both the mean cortisol and fasting cortisol were found to be significantly lower in CT vs. the control, whilst in AN hypercortisolism is characteristic [85]. As hypoglycaemia is not the driving force for endocrine adaptations in CT, as it appears to be in AN, because being underweight is not a result of reduced calorie intake, but of an altered metabolism yet to be clearly defined, this observation is consistent with the findings commented on above.

## 4. Discussion

Alterations in the endocrinology of underweight have been long known and have even been, in the case of secondary amenorrhea following the down-regulation of the HPG axis, a diagnostic criterion for anorexia nervosa in the past.

When reviewing the research on hormones in underweight, it has to be noted that male individuals are, by far, underrepresented in these studies. Also, patients in early puberty have mostly not been included, especially in interventional studies, resulting in a lack of knowledge as to which long-term effects on bone structure and reproductive health could be reduced by the application of, for example, transdermal oestrogen.

Many studies have been of limited power, especially when looking at the less commonly examined hormones.

Among the pituitary hormones that have been subject to relatively broad investigation, apart from the aforementioned substitution of sex hormones, there are limited options concerning the hormonal treatment of being underweight. Looking at the hormones regulating appetite, based on smaller studies, there is some indication that metreleptin can improve outcomes in AN.

Regarding the comparison of CT and AN, hormone levels can help to distinguish between these two conditions if in doubt. With the changing diagnostic criteria (amenorrhea not being a criterion for AN anymore), a shift in prevalence rates could be observed, which could lead to the question now of how many cases of CT were counted as AN. The endocrine differences summed up here can prevent possible confusion between the two.

## 5. Conclusions

In anorexia nervosa, semi-starvation leads to multiple endocrine alterations, most of which are adaptive and reversible with weight restoration. Therefore, there is limited need for medical intervention. Thyroid hormones will, in most cases, readjust, as well as gonadal hormones; however, in order to prevent further bone loss, transcutaneous oestrogen and oral gestagen are advisable on top of vitamin D and calcium supplementation.

Constitutional thinness shows clinical similarities to anorexia nervosa as in affected bone health, but also clearly distinctive endocrine findings, most importantly those for fT3 and cortisol. The ratio of resting metabolic rate and fat-free mass is a promising field of research in this context.

## Figures and Tables

**Table 1:** Pituitary hormones.

Hormone	Target(s)	Function
ACTH	Adrenals	Stimulates the adrenal gland to produce a hormone called cortisol. ACTH is also known as corticotrophin.
TSH	Thyroid	Stimulates the thyroid gland to secrete its own hormone, which is called thyroxine. TSH is also known as thyrotrophin.
LH and FSH	Ovaries (women) Testes (men)	Controls reproductive functioning and sexual characteristics. Stimulates the ovaries to produce oestrogen and progesterone and the testes to produce testosterone and sperm. LH and FSH are known collectively as gonadotrophins. LH is also referred to as interstitial cell-stimulating hormone (ICSH) in males.
PRL	Breasts	Stimulates the breasts to produce milk. This hormone is secreted in large amounts during pregnancy and breastfeeding, but is present at all times in both men and women.
GH	All cells in the body	Stimulates growth and repair. Research is currently being carried out to identify the functions of GH in adult life.
MSH		Exact role in humans is unknown.
ADH	Kidneys	Controls the blood fluid and mineral levels in the body by affecting water retention by the kidneys. This hormone is also known as vasopressin or arginine vasopressin (AVP).
Oxytocin	Uterus, breasts	Affects uterine contractions in pregnancy and birth and subsequent release of breast milk.

**Table 2:** Summary of distinct comparable parameters in AN, CT, and a control group.

Parameters	Comparison
Fat-free mass (FFM)	AN = CT < Control
Bone mass density (BMD)	AN = CT < Control
Fat mass (FM)	AN < CT < Control
Body mass index (BMI)	AN < CT < Control
Leptin (fasting and 24 h mean)	AN < CT < Control
Resting metabolic rate (RMR)	AN < CT < Control
Total energy intake	AN < CT = Control
Estradiol	AN < CT = Control
FSH and LH	AN < CT = Control
IGF-1	AN < CT = Control
GH (fasting and mean)	AN > CT = Control
fT3	AN < Control < CT
Mean cortisol	AN > Control > CT
Fasting cortisol	AN > Control > CT

## Data Availability

Data sharing not applicable. No new data were created or analysed in this study. Data sharing is not applicable to this article.

## References

[1] Misra M., Miller K.K., Almazan C., Ramaswamy K., Lapcharoensap W., Worley M., Neubauer G., Herzog D.B., Klibanski A. (2004). Alterations in cortisol secretory dynamics in adolescent girls with anorexia nervosa and effects on bone metabolism. J. Clin. Endocrinol. Metab..

[2] Estour B., Germain N., Diconne E., Frere D., Cottet-Emard J.M., Carrot G., Lang F., Galusca B. (2010). Hormonal profile heterogeneity and short-term physical risk in restrictive anorexia nervosa. J. Clin. Endocrinol. Metab..

[3] Aschettino-Manevitz D.L., Ornstein R.M., Meyer Sterling W., Kohn N., Fisher M. (2012). Triiodothyronine (T3) and metabolic rate in adolescents with eating disorders: Is there a correlation?. Eat. Weight Disord..

[4] Swenne I., Rosling A. (2010). Do thyroid hormones mediate the effects of starvation on mood in adolescent girls with eating disorders?. Psychoneuroendocrinology.

[5] Swenne I., Stridsberg M., Thurfjell B., Rosling A. (2009). Triiodothyronine is an indicator of nutritional status in adolescent girls with eating disorders. Horm. Res..

[6] Kaplan R. (1998). Thyroxine abuse. Aust. N. Z. J. Psychiatry.

[7] Boyar R.M., Hellman L.D., Roffwarg H., Katz J., Zumoff B., O’Connor J., Bradlow H.L., Fukushima D.K. (1977). Cortisol secretion and metabolism in anorexia nervosa. N. Engl. J. Med..

[8] Stoving R.K. (2019). Mechanisms in endocrinology: Anorexia nervosa and endocrinology: A clinical update. Eur. J. Endocrinol..

[9] Misra M., Klibanski A. (2011). The neuroendocrine basis of anorexia nervosa and its impact on bone metabolism. Neuroendocrinology.

[10] Grinspoon S., Thomas L., Miller K., Pitts S., Herzog D., Klibanski A. (2001). Changes in regional fat redistribution and the effects of estrogen during spontaneous weight gain in women with anorexia nervosa. Am. J. Clin. Nutr..

[11] Schorr M., Miller K.K. (2017). The endocrine manifestations of anorexia nervosa: Mechanisms and management. Nat. Rev. Endocrinol..

[12] Van Binsbergen C.J., Coelingh Bennink H.J., Odink J., Haspels A.A., Koppeschaar H.P. (1990). A comparative and longitudinal study on endocrine changes related to ovarian function in patients with anorexia nervosa. J. Clin. Endocrinol. Metab..

[13] Miller K.K., Grinspoon S., Gleysteen S., Grieco K.A., Ciampa J., Breu J., Herzog D.B., Klibanski A. (2004). Preservation of neuroendocrine control of reproductive function despite severe undernutrition. J. Clin. Endocrinol. Metab..

[14] Jacoangeli F., Masala S., Staar Mezzasalma F., Fiori R., Martinetti A., Ficoneri C., Novi B., Pierangeli S., Marchetti G., Simonetti G. (2006). Amenorrhea after weight recover in anorexia nervosa: Role of body composition and endocrine abnormalities. Eat. Weight Disord..

[15] Latzer I.T., Kidron-Levy H., Stein D., Levy A.E., Yosef G., Ziv-Baran T., Dubnov-Raz G. (2019). Predicting menstrual recovery in adolescents with anorexia nervosa using body fat percent estimated by bioimpedance analysis. J. Adolesc. Health.

[16] Misra M., Katzman D., Miller K.K., Mendes N., Snelgrove D., Russell M., Goldstein M.A., Ebrahimi S., Clauss L., Weigel T. (2011). Physiologic estrogen replacement increases bone density in adolescent girls with anorexia nervosa. J. Bone Min. Res..

[17] Misra M., Katzman D.K., Estella N.M., Eddy K.T., Weigel T., Goldstein M.A., Miller K.K., Klibanski A. (2013). Impact of physiologic estrogen replacement on anxiety symptoms, body shape perception, and eating attitudes in adolescent girls with anorexia nervosa: Data from a randomized controlled trial. J. Clin. Psychiatry.

[18] Golden N.H., Kreitzer P., Jacobson M.S., Chasalow F.I., Schebendach J., Freedman S.M., Shenker I.R. (1994). Disturbances in growth hormone secretion and action in adolescents with anorexia nervosa. J. Pediatr..

[19] Keeler J.L., Robinson L., Keeler-Schäffeler R., Dalton B., Treasure J., Himmerich H. (2022). Growth factors in anorexia nervosa: A systematic review and meta-analysis of cross-sectional and longitudinal data. World J. Biol. Psychiatry.

[20] Hotta M., Fukuda I., Sato K., Hizuka N., Shibasaki T., Takano K. (2000). The relationship between bone turnover and body weight, serum insulin-like growth factor (IGF) I, and serum IGF-binding protein levels in patients with anorexia nervosa. J. Clin. Endocrinol. Metab..

[21] Argente J., Caballo N., Barrios V., Munoz M.T., Pozo J., Chowen J.A., Hernandez M. (1997). Disturbances in the growth hormone-insulin-like growth factor axis in children and adolescents with different eating disorders. Horm. Res..

[22] Fazeli P.K., Lawson E.A., Faje A.T., Eddy K.T., Lee H., Fiedorek F.T., Breggia A., Gaal I.M., DeSanti R., Klibanski A. (2018). Treatment with a Ghrelin Agonist in Outpatient Women With Anorexia Nervosa: A Randomized Clinical Trial. J. Clin. Psychiatry.

[23] Bahia A., Chu E.S., Mehler P.S. (2011). Polydipsia and hyponatremia in a woman with anorexia nervosa. Int. J. Eat. Disord..

[24] Puckett L., Grayeb D., Khatri V., Cass K., Mehler P. (2021). A comprehensive review of complications and new findings associated with anorexia nervosa. J. Clin. Med..

[25] Spasovski G., Vanholder R., Allolio B., Annane D., Ball S., Bichet D., Decaux G., Fenske W., Hoorn E.J., Ichai C. (2014). Clinical Practice Guideline Clinical practice guideline on diagnosis and treatment of hyponatraemia. Nephrol. Dial. Transplant..

[26] Lawson E.A., Donoho D.A., Blum J.I., Meenaghan E.M., Misra M., Herzog D.B., Sluss P.M., Miller K.K., Klibanski A. (2011). Decreased nocturnal oxytocin levels in anorexia nervosa are associated with low bone mineral density and fat mass. J. Clin. Psychiatry.

[27] Lawson E.A., Holsen L.M., Santin M., DeSanti R., Meenaghan E., Eddy K.T., Herzog D.B., Goldstein J.M., Klibanski A. (2013). Postprandial oxytocin secretion is associated with severity of anxiety and depressive symptoms in anorexia nervosa. J. Clin. Psychiatry.

[28] Culbert K.M., Racine S.E., Klump K.L. (2016). Hormonal Factors and Disturbances in Eating Disorders. Curr. Psychiatry Rep..

[29] Schorr M., Marengi D.A., Pulumo R.L., Yu E., Eddy K.T., Klibanski A., Miller K.K., Lawson E.A. (2017). Oxytocin and Its Relationship to Body Composition, Bone Mineral Density, and Hip Geometry across the Weight Spectrum. J. Clin. Endocrinol. Metab..

[30] Faje A.T., Karim L., Taylor A., Lee H., Miller K.K., Mendes N., Meenaghan E., Goldstein M.A., Bouxsein M.L., Misra M. (2013). Adolescent girls with anorexia nervosa have impaired cortical and trabecular microarchitecture and lower estimated bone strength at the distal radius. J. Clin. Endocrinol. Metab..

[31] Faje A.T., Fazeli P.K., Miller K.K., Katzman D.K., Ebrahimi S., Lee H., Mendes N., Snelgrove D., Meenaghan E., Misra M. (2014). Fracture risk and areal bone mineral density in adolescent females with anorexia nervosa. Int. J. Eat. Disord..

[32] Bredella M.A., Misra M., Miller K.K., Klibanski A., Gupta R. (2008). Trabecular structure analysis of the distal radius in adolescent patients with anorexia nervosa using ultra high resolution flat panel based volume CT. J. Musculoskelet. Neuronal Interact..

[33] Lawson E.A., Miller K.K., Bredella M.A., Phan C., Misra M., Meenaghan E., Rosenblum L., Donoho D., Gupta R., Klibanski A. (2010). Hormone predictors of abnormal bone microarchitecture in women with anorexia nervosa. Bone.

[34] Misra M., Klibanski A. (2011). Bone metabolism in adolescents with anorexia nervosa. J. Endocrinol. Investig..

[35] Fazeli P.K., Klibanski A. (2018). Effects of Anorexia Nervosa on Bone Metabolism. Endocr. Rev..

[36] Robinson L., Micali N., Misra M. (2017). Eating disorders and bone metabolism in women. Curr. Opin. Pediatr..

[37] Tomoda K., Yoshikawa M., Itoh T., Tamaki S., Fukuoka A., Komeda K., Kimura H. (2007). Elevated circulating plasma adiponectin in underweight patients with COPD. Chest.

[38] Tural U., Iosifescu D.V. (2022). Adiponectin in anorexia nervosa and its modifiers: A meta-regression study. Int. J. Eat. Disord..

[39] Tyszkiewicz-Nwafor M., Jowik K., Paszynska E., Dutkiewicz A., Słopien A., Dmitrzak-Weglarz M. (2022). Expression of immune-related proteins and their association with neuropeptides in adolescent patients with anorexia nervosa. Neuropeptides.

[40] Tyszkiewicz-Nwafor M., Slopien A., Dmitrzak-Węglarz M., Rybakowski F. (2019). Adiponectin and resistin in acutely ill and weight-recovered adolescent anorexia nervosa: Association with psychiatric symptoms. World J. Biol. Psychiatry.

[41] Uzum A.K., Aydin M.M., Tutuncu Y., Omer B., Kiyan E., Alagol F. (2014). Serum ghrelin and adiponectin levels are increased but serum leptin level is unchanged in low weight Chronic Obstructive Pulmonary Disease patients. Eur. J. Intern. Med..

[42] Iwahashi H., Funahashi T., Kurokawa N., Sayama K., Fukuda E., Okita K., Imagawa A., Yamagata K., Shimomura I., Miyagawa J.I. (2003). Plasma adiponectin levels in women with anorexia nervosa. Horm. Metab. Res..

[43] Housova J., Anderlova K., Krizova J., Haluzikova D., Kremen J., Kumstyrova T., Papezova H., Haluzik M. (2005). Serum adiponectin and resistin concentrations in patients with restrictive and binge/purge form of anorexia nervosa and bulimia nervosa. J. Clin. Endocrinol. Metab..

[44] Pannacciulli N., Vettor R., Milan G., Granzotto M., Catucci A., Federspil G., De Giacomo P., Giorgino R., De Pergola G. (2003). Anorexia nervosa is characterized by increased adiponectin plasma levels and reduced nonoxidative glucose metabolism. J. Clin. Endocrinol. Metab..

[45] Tang N., Zhang X., Chen D., Li Z. (2021). The controversial role of adiponectin in appetite regulation of animals. Nutrients.

[46] Zhang Y., Proenca R., Maffei M., Barone M., Leopold L., Friedman J.M. (1994). Positional cloning of the mouse obese gene and its human homologue. Nature.

[47] Hebebrand J., van der Heyden J., Devos R., Kopp W., Herpertz S., Remschmidt H., Herzog W. (1995). Plasma concentrations of obese protein in anorexia nervosa. Lancet.

[48] Herpertz S., Wagner R., Albers N., Blum W.F., Pelz B., Langkafel M., Kopp W., Henning A., Oberste-Berghaus C., Mann K. (1998). Circadian plasma leptin levels in patients with anorexia nervosa: Relation to insulin and cortisol. Horm. Res..

[49] Haluzik M., Papezova M., Nedvidkova J., Kabrt J. (1999). Serum leptin levels in patients with anorexia nervosa before and after partial refeeding, relationships to serum lipids and biochemical nutritional parameters. Physiol. Res..

[50] Exner C., Hebebrand J., Remschmidt H., Wewetzer C., Ziegler A., Herpertz S., Schweiger U., Blum W.F., Preibisch G., Heldmaier G. (2000). Leptin suppresses semi-starvation induced hyperactivity in rats: Implications for anorexia nervosa. Mol. Psychiatry.

[51] Hebebrand J., Exner C., Hebebrand K., Holtkamp C., Casper R.C., Remschmidt H., Herpertz-Dahlmann B., Klingenspor M. (2003). Hyperactivity in patients with anorexia nervosa and in semistarved rats: Evidence for a pivotal role of hypoleptinemia. Physiol. Behav..

[52] Dalle Grave R., Calugi S., Marchesini G. (2008). Compulsive exercise to control shape or weight in eating disorders: Prevalence, associated features, and treatment outcome. Compr. Psychiatry.

[53] Davis C., Katzman D.K., Kirsh C. (1999). Compulsive physical activity in adolescents with anorexia nervosa: A psychobehavioral spiral of pathology. J. Nerv. Ment. Dis..

[54] El Ghoch M., Calugi S., Pellegrini M., Milanese C., Busacchi M., Battistini N.C., Bernabe J., Dalle Grave R. (2013). Measured physical activity in anorexia nervosa: Features and treatment outcome. Int. J. Eat. Disord..

[55] Hechler T., Rieger E., Touyz S., Beumont P., Plasqui G., Westerterp K. (2008). Physical activity and body composition in outpatients recovering from anorexia nervosa and healthy controls. Adapt. Phys. Act. Q..

[56] Gummer R., Giel K.E., Schag K., Resmark G., Junne F.P., Becker S., Zipfel S., Teufel M. (2015). High Levels of Physical Activity in Anorexia Nervosa: A Systematic Review. Eur. Eat. Disord. Rev..

[57] Kopp W., Blum W.F., von Prittwitz S., Ziegler A., Lubbert H., Emons G., Herzog W., Herpertz S., Deter H.C., Remschmidt H. (1997). Low leptin levels predict amenorrhea in underweight and eating disordered females. Mol. Psychiatry.

[58] Matejek N., Weimann E., Witzel C., Molenkamp G., Schwidergall S., Bohles H. (1999). Hypoleptinaemia in patients with anorexia nervosa and in elite gymnasts with anorexia athletica. Int. J. Sports Med..

[59] Antel J., Tan S., Grabler M., Ludwig C., Lohkemper D., Brandenburg T., Barth N., Hinney A., Libuda L., Remy M. (2022). Rapid amelioration of anorexia nervosa in a male adolescent during metreleptin treatment including recovery from hypogonadotropic hypogonadism. Eur. Child Adolesc. Psychiatry.

[60] Kim Y., Hersch J., Bodell L.P., Schebendach J., Hildebrandt T., Walsh B.T., Mayer L.E.S. (2021). The association between leptin and weight maintenance outcome in anorexia nervosa. Int. J. Eat. Disord..

[61] Milos G., Antel J., Kaufmann L.K., Barth N., Koller A., Tan S., Wiesing U., Hinney A., Libuda L., Wabitsch M. (2020). Short-term metreleptin treatment of patients with anorexia nervosa: Rapid on-set of beneficial cognitive, emotional, and behavioral effects. Transl. Psychiatry.

[62] Gradl-Dietsch G., Milos G., Wabitsch M., Bell R., Tschöpe F., Antel J., Hebebrand J. (2023). Rapid Emergence of Appetite and Hunger Resulting in Weight Gain and Improvement of Eating Disorder Symptomatology during and after Short-Term Off-Label Metreleptin Treatment of a Patient with Anorexia Nervosa. Obes. Facts.

[63] Hebebrand J., Milos G., Wabitsch M., Teufel M., Führer D., Bühlmeier J., Libuda L., Ludwig C., Antel J. (2019). Clinical Trials Required to Assess Potential Benefits and Side Effects of Treatment of Patients with Anorexia Nervosa with Recombinant Human Leptin. Front. Psychol..

[64] Achamrah N., Coëffier M., Déchelotte P. (2016). Physical activity in patients with anorexia nervosa. Nutr. Rev..

[65] Gianini L.M., Klein D.A., Call C., Walsh B.T., Wang Y., Wu P., Attia E. (2016). Physical activity and post-treatment weight trajectory in anorexia nervosa. Int. J. Eat. Disord..

[66] Rizk M., Mattar L., Kern L., Berthoz S., Duclos J., Viltart O., Godart N. (2020). Physical Activity in Eating Disorders: A Systematic Review. Nutrients.

[67] Toutain M., Gauthier A., Leconte P. (2022). Exercise therapy in the treatment of anorexia nervosa: Its effects depending on the type of physical exercise—A systematic review. Front. Psychiatry.

[68] Dittmer N., Voderholzer U., Mönch C., Cuntz U., Jacobi C., Schlegl S. (2020). Efficacy of a specialized group intervention for compulsive exercise in inpatients with anorexia nervosa: A randomized controlled trial. Psychother. Psychosom..

[69] Harty R.F., Pearson P.H., Solomon T.E., McGuigan J.E. (1991). Cholecystokinin, vasoactive intestinal peptide and peptide histidine methionine responses to feeding in anorexia nervosa. Regul. Pept..

[70] Pirke K.M., Kellner M.B., Friess E., Krieg J.C., Fichter M.M. (1994). Satiety and cholecystokinin. Int. J. Eat. Disord..

[71] Geracioti T.D., Liddle R.A., Altemus M., Demitrack M.A., Gold P.W. (1992). Regulation of appetite and cholecystokinin secretion in anorexia nervosa. Am. J. Psychiatry.

[72] Cuntz U., Enck P., Fruhauf E., Lehnert P., Riepl R.L., Fichter M.M., Otto B. (2013). Cholecystokinin revisited: CCK and the hunger trap in anorexia nervosa. PLoS ONE.

[73] Misra M., Klibanski A. (2014). Anorexia nervosa and bone. J. Endocrinol..

[74] Tam F.I., Seidel M., Boehm I., Ritschel F., Bahnsen K., Biemann R., Weidner K., Roessner V., Ehrlich S. (2020). Peptide YY(3-36) concentration in acute- and long-term recovered anorexia nervosa. Eur. J. Nutr..

[75] Becker K.R., Mancuso C., Dreier M.J., Asanza E., Breithaupt L., Slattery M., Plessow F., Micali N., Thomas J.J., Eddy K.T. (2021). Ghrelin and PYY in low-weight females with avoidant/restrictive food intake disorder compared to anorexia nervosa and healthy controls. Psychoneuroendocrinology.

[76] Otto B., Cuntz U., Otto C., Heldwein W., Riepl R.L., Tschop M.H. (2007). Peptide YY release in anorectic patients after liquid meal. Appetite.

[77] Pfluger P.T., Kampe J., Castaneda T.R., Vahl T., D’Alessio D.A., Kruthaupt T., Benoit S.C., Cuntz U., Rochlitz H.J., Moehlig M. (2007). Effect of human body weight changes on circulating levels of peptide YY and peptide YY3-36. J. Clin. Endocrinol. Metab..

[78] Nakahara T., Kojima S., Tanaka M., Yasuhara D., Harada T., Sagiyama K.-I., Muranaga T., Nagai N., Nakazato M., Nozoe S.-I. (2007). Incomplete restoration of the secretion of ghrelin and PYY compared to insulin after food ingestion following weight gain in anorexia nervosa. J. Psychiatr. Res..

[79] Otto B., Cuntz U., Fruehauf E., Wawarta R., Folwaczny C., Riepl R.L., Heiman M.L., Lehnert P., Fichter M., Tschop M. (2001). Weight gain decreases elevated plasma ghrelin concentrations of patients with anorexia nervosa. Eur. J. Endocrinol..

[80] Dostalova I., Haluzik M. (2009). The role of ghrelin in the regulation of food intake in patients with obesity and anorexia nervosa. Physiol. Res..

[81] Krsek M., Rosicka M., Papezova H., Krizova J., Kotrlikova E., Haluz’k M., Justova V., Lacinova Z., Jarkovska Z. (2003). Plasma ghrelin levels and malnutrition: A comparison of two etiologies. Eat. Weight Disord..

[82] Otto B., Tschop M., Fruhauf E., Heldwein W., Fichter M., Otto C., Cuntz U. (2005). Postprandial ghrelin release in anorectic patients before and after weight gain. Psychoneuroendocrinology.

[83] Hotta M., Ohwada R., Akamizu T., Shibasaki T., Takano K., Kangawa K. (2009). Ghrelin increases hunger and food intake in patients with restricting-type anorexia nervosa: A pilot study. Endocr. J..

[84] Hotta M., Ohwada R., Akamizu T., Shibasaki T., Kangawa K. (2012). Therapeutic potential of ghrelin in restricting-type anorexia nervosa. Methods Enzymol..

[85] Estour B., Marouani N., Sigaud T., Lang F., Fakra E., Ling Y., Diamonde A., Minnion J.S., Galusca B., Germain N. (2017). Differentiating constitutional thinness from anorexia nervosa in DSM 5 era. Psychoneuroendocrinology.

[86] Pehlivantürk Kızılkan M., Akgül S., Derman O., Kanbur N. (2018). Bone mineral density comparison of adolescents with constitutional thinness and anorexia nervosa. J. Pediatr. Endocrinol. Metab..

[87] Galusca B., Germain N., Estour B. (2009). Bone abnormalities in constitutional thinness. Br. J. Nutr..

[88] Bailly M., Boscaro A., Pereira B., Courteix D., Germain N., Galusca B., Boirie Y., Thivel D., Verney J. (2021). Underweight but not underfat: Is fat-free mass a key factor in constitutionally thin women?. Eur. J. Clin. Nutr..

[89] Bailly M., Boscaro A., Pereira B., Féasson L., Boirie Y., Germain N., Galusca B., Courteix D., Thivel D., Verney J. (2021). Is constitutional thinness really different from anorexia nervosa? A systematic review and meta-analysis. Rev. Endocr. Metab. Disord..

[90] Bailly M., Germain N., Galusca B., Courteix D., Thivel D., Verney J. (2020). Definition and diagnosis of constitutional thinness: A systematic review. Br. J. Nutr..

[91] Tolle V., Kadem M., Bluet-Pajot M.T., Frere D., Foulon C., Bossu C., Dardennes R., Mounier C., Zizzari P., Lang F. (2003). Balance in ghrelin and leptin plasma levels in anorexia nervosa patients and constitutionally thin women. J. Clin. Endocrinol. Metab..

